# Bridging the gap between the laboratory and the clinic for patients with sarcopenia

**DOI:** 10.1007/s10522-018-09793-z

**Published:** 2018-12-27

**Authors:** Miles D. Witham

**Affiliations:** AGE Research Group, NIHR Newcastle Biomedical Research Centre, Biomedical Research Building, Campus for Ageing and Vitality, Newcastle, NE4 5PL UK

**Keywords:** Sarcopenia, Clinical research, Trials, Translational medicine

## Abstract

Sarcopenia—the age-related loss of skeletal muscle mass and strength—is a major public health issue. Sarcopenia is associated with an increased risk of falls, disability, dependency, institutionalization, hospital stay and early death. Finding interventions to stabilize, reverse or prevent sarcopenia is therefore a key goal for clinical ageing research. If patients are to eventually benefit from discovery science on ageing skeletal muscle, we need to build a translational pipeline that facilitates progress from laboratory science and epidemiology, through feasibility testing to early-phase, and eventually late-phase clinical trials. A number of barriers need to be overcome to make this pipeline work—in particular challenges around identifying people with sarcopenia in routine clinical practice, ensuring that we study patients with clearly defined sarcopenia rather than related conditions such as functional impairment, developing capacity to run trials for older people, and selecting trial outcomes of relevance to older people with multimorbidity. A further key point is that interventions should ideally have pleiotropic actions—i.e. beneficial actions across multiple organ systems, rather than treating sarcopenia alone. Such pleiotropic interventions may be the only way to avoid the perils of polypharmacy and drug interactions that bedevil care for many older people. Maximising the potential for scientific discoveries in the biology of ageing muscle to improve health requires that discovery scientists, translational clinical scientists and clinicians come together to exchange findings and shape each others ideas within a shared culture.

## Introduction

Sarcopenia is a relative newcomer in the diagnostic pantheon compared with many illnesses that accompany advanced age. Although the loss of muscle mass and strength that define sarcopenia were recognised in ancient times, the modern use of the term dates back only to the late 1980s (Rosenberg 1997), and the use of sarcopenia as a diagnostic term is even more recent, receiving an ICD-10 code as recently as 2016. The definition continues to undergo refinement (Cruz-Jentoft et al. 2010, 2018; Studenski et al. 2014). Despite this, considerable progress is being made in understanding both the epidemiology and pathophysiology of sarcopenia (Sayer et al. 2013), and in common with many other diseases such as dementia that are more common with advanced age, there is now a realisation that sarcopenia may not be an inevitable consequence of ageing.

It is now clear that sarcopenia is common, affecting 5–10% of the general population aged over 65, but up to 50% of nursing home residents (Mayhew et al. 2018; Shen et al. 2018). Sarcopenia is associated with a wide range of serious consequences for older people—falls, hospitalisation, longer length of stay in hospital, a greater need for care including nursing home care, and finally earlier death (Beaudart et al. 2017). Sarcopenia is costly—the estimated cost to the UK National Health Service is £2.5 billion per year (Pinedo-Villanueva et al. 2018). Sarcopenia is therefore an important condition for us to understand, diagnose and treat. Although there is much still to learn about the aetiology and pathophysiology of sarcopenia, a range of culprit biological systems are under active investigation, including pertubations in hormonal systems, chronic inflammation, mitochondrial function, and networks of adipokines and myokines (Morley 2016).

These insights are starting to suggest a wide range of potential therapeutic targets that will require testing in clinical studies including trials. Doing so will not be straightforward, and this paper discusses both the barriers to conducting such trials, and gives some suggestions for how we can make progress so that the burgeoning insights from discovery science make their way successfully into clinical practice.

## What are the key barriers to translating discovery science into clinical treatments for sarcopenia?

### General considerations for trials for older people

#### Signal to noise ratio

Trials in older people are difficult, and these difficulties have deterred investigators in many other organ-specific conditions from designing and delivering clinical trials for older people. To detect a statistically significant treatment effect, trials need to recruit sufficient participants to differentiate the effect (signal) from the noise (variation). For older people, this signal to noise ratio is low—most biological measures are more heterogeneous in old age that in younger people, and the trajectory followed by older people is rarely stable. Illness, and the recovery from illness, are common in older people. This is particularly the case for sarcopenia, which is frequently accompanied by multiple other conditions (Volpato et al. 2014). A related issue is that older people with multiple conditions are more likely to die or become too ill to continue during a trial; failure to undergo follow up measurements reduces trial power and requires larger initial sample sizes.

Attempts to exclude those with comorbidity are counterproductive; not only does this approach hamper trial recruitment; it leads to trial populations which are unrepresentative of the broader population (McMurdo et al. 2005). This lack of generalisability for trial results is a major barrier to the uptake of trial findings into clinical practice—clinicians conclude that the trial participants “aren’t the patients we care for”. Heart failure, hypertension, diabetes and cancer are just some of the disease areas that have previously been affected by this lack of generalisability of trial results (Cherubini et al. 2011; Conroy et al. 2018; Cruz-Jentoft et al. 2013; Townsley et al. 2005). Some of the above problems can be overcome by increasing initial sample sizes, but the additional time and cost, large number of adverse events and dropouts (usually unrelated to the trial intervention) have made trials for older people unattractive to both commercial and academic investigators to date.

#### Multimorbidity and the choice of outcomes

Multimorbidity is the norm for older people (Barnett et al. 2012), yet most clinical medical research focusses on single conditions, often affecting a single organ. Such an approach is once again at variance with the lived experience of patients, but is also at variance with the needs of clinicians and policymakers. Clinical guidelines—often the endpoint of a long research process—are mostly based on single conditions, with little attention paid to how such guidelines overlap or conflict with each other. For older people with multiple conditions, applying multiple sets of guidelines leads to an intolerable treatment burden (Buffel du Vaure et al. 2016), polypharmacy (with a danger of multiple side effects and drug interactions) (Scottish Government 2018) and frankly conflicting advice (Hughes et al. 2013).

If we are to avoid pursuing research that ends with such an unhelpful evidence base, we need to ensure that trials for sarcopenia are designed differently. This applies as much to early-phase translational work as to later-phase trials. It is often argued that homogeneity and a lack of confounding factors are important when attempting to demonstrate proof of concept or efficacy (Kent and Kitsios 2009). This may indeed make it easier to demonstrate efficacy, and avoids the issue that other diagnoses may be more important drivers of impairment for some individuals than sarcopenia. However, if a potential treatment cannot show proof-of-concept in a group of older people with multiple other conditions, there is little point in taking the intervention forward for testing in large-scale, later-phase trials.

Although selecting the right population to study is important, selecting the right outcomes is also key. Research into single organ conditions has tended to select outcomes that focus only on the function of that organ, missing both potential harms and the overall balance of risk and benefit to patients. Such an approach is worsened by the use of surrogate outcomes—intermediate measures that are thought to be related to clinically important outcomes. Use of such outcomes requires careful validation and interpretation to avoid drawing misleading conclusions. The example of glycosylated haemoglobin (HbA1c) in diabetes mellitus is instructive in this regard. Although HbA1c levels are predictive of microvascular outcomes (such as retinopathy and neuropathy), they bear little relation to macrovascular outcomes (such as heart disease and stroke). Furthermore, a narrow focus on reducing HbA1c fails to capture other important harms, including hypoglycaemia and its attendant consequences such as falls and cognitive impairment. Despite these limitations, ability to reduce HbA1c has been used as a regulatory approval criterion for new diabetes medications for many years, and many trials in diabetes still focus on whether interventions reduce HbA1c, yet this approach gives a very incomplete picture of the overall harms and benefits of interventions. It is only in the last few years that regulators have started to demand large trials measuring cardiovascular endpoints for new diabetes medications (Cefalu et al. 2018).

The situation is similar for studies in the field of sarcopenia. Many studies focus on muscle mass, yet increasing muscle mass without an improvement in strength or performance is unlikely to be relevant to older people. Muscle strength is a much better predictor of the ability to perform activities of daily living—yet even this approach does not enable the potential downsides of therapy (which may affect systems outwith the musculoskeletal system) to be captured and compared. Physical function and quality of life are outcomes that older people value most (Roberts et al. 1994); it therefore follows that these are the outcomes that any intervention should be attempting to improve—and thus should be key outcomes in clinical trials for sarcopenia interventions.

#### Physical, social and cognitive impairment

Recruiting older people to clinical trials takes additional time and specific skills on the part of investigators and research teams. As argued above, recruiting healthy older people to trials does not lead to a representative trial population. However, many trials are based in large hospitals, often with difficult transport links and long walks required to reach the study centre after arriving at the hospital. Hospitals are busy, confusing places even for those with intact cognition and no impairment of vision and hearing. Many older people find that their social network has become eroded through death and incapacity, and thus lack people who can support them to attend study visits. Once at the study centre, older people may have to deal with long, dense documents such as study information sheets, and endure long study visits of several hours. All of these factors act as potential disincentives to trial participation, but also provide investigators with potential ways to improve the trial experience for older people by providing taxi transport, siting research centres within easy walking or wheelchair distance, and minimising both study visit duration and the number of outcomes measured.

## Specific issues in the design and execution of sarcopenia trials

### Studies need to recruit populations with a defined diagnosis of sarcopenia

**Fig. 1:**
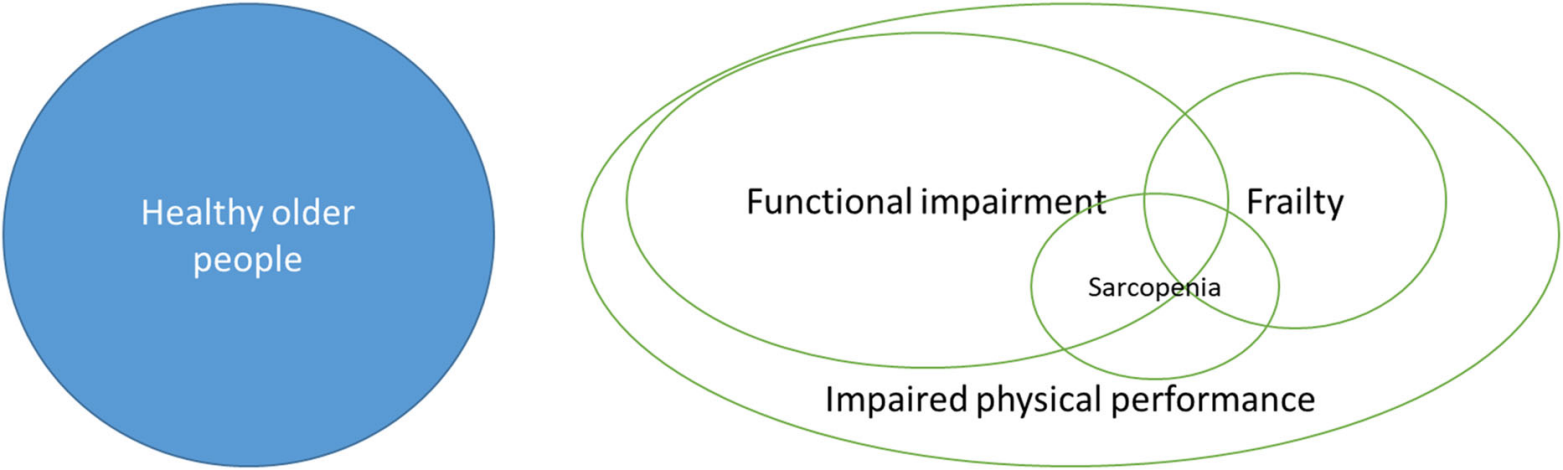
Difference in target populations for muscle studies in older people

Sarcopenia as an entity is a relatively recent arrival in the research and clinical landscape, and so it is perhaps unsurprising that most clinical studies to date have not been conducted in populations with sarcopenia. Whilst there is clearly overlap between sarcopenia and other, more commonly measured constructs (e.g. frailty and functional impairment) (Fig. 1), the degree of overlap is not usually clear from published reports. This matters, because interventions that might work in the specific disease state of sarcopenia might do nothing in healthy older people, or in those with for example preserved muscle mass but impaired function. The converse is also true. At the very least, effects might be diluted if a study population has only a proportion with sarcopenia; such studies would then require larger number of participants to demonstrate differences between intervention and control groups.

### Finding people with sarcopenia is challenging

The diagnosis of sarcopenia is not often made in current clinical practice—even by geriatricians, who specialise in the care of older people. A diagnostic code (ICD-10) code did not exist for coding sarcopenia until late 2016, and even when sarcopenia is diagnosed, it is poorly recorded in both paper and electronic records. A vicious cycle currently exists within clinical practice; there is a lack of interventions to prevent or reverse sarcopenia beyond resistance training, thus there is little added value to clinicians in making or recording the diagnosis as doing so does not change treatment. Although measuring grip strength, walk speed or chair stand time is simple and quick to do in clinical practice, the need to measure muscle mass to make a diagnosis of sarcopenia is a barrier to making the diagnosis; imaging techniques such as DEXA, CT or MRI scanning are expensive and non-portable; use of techniques such as bioimpedance measurement require careful validation of measurement tools in specific populations (Reiter et al. 2019) to generate useful results. The most recent sarcopenia diagnostic guidelines recognise this barrier to diagnosis and allow a diagnosis of ‘probable sarcopenia’ (Cruz-Jentoft et al. 2018) based solely on low muscle strength, but this approach risks altering the target population for trials and makes comparison with studies using a strength plus mass-based diagnosis difficult.

For research teams, this lack of routine diagnosis and recording means that it is necessary to screen large numbers of older people for low muscle mass and strength when conducting clinical research in sarcopenia (Marzetti et al. 2018). Efficient systems to find people with sarcopenia are therefore needed and potential solutions are discussed later in this article. Further disincentives exist for commercial partners to engage in sarcopenia research; the diagnosis cannot currently be used to base marketing authorisation for new drugs, and those seeking to develop new agents are forced to use other conditions (e.g. falls or hip fracture) as endpoints to allow marketing authorisation. Work is ongoing between research teams and regulators including the US Food and Drug Administration and the European Medicines Agency (Le Lain et al. 2017) to define endpoints for sarcopenia trials that would enable regulatory approval of new therapeutics.

A related, but distinct set of challenges face researchers who wish to prevent the development of sarcopenia—an approach that is likely to be essential if we are to relieve the burden of sarcopenia at the population level. More work is needed on factors that predict the future onset of sarcopenia to enable selection of individuals most likely to benefit from prevention, and similar considerations to those discussed above apply to the choice of outcome measures. The need for short-term biomarker predictors of response is arguably even greater for prevention trials, as interventions to prevent sarcopenia may require to be given for very long periods of time (years to decades) if the life-course trajectory of skeletal muscle decline is to be usefully modified (Sayer et al. 2013).

## Steps to bridging the gap between laboratory science and clinical practice

How then can we develop programmes of research in sarcopenia that mitigate or circumvent the issues described above, and that enable faster, smoother translation of the wealth of discovery science findings into new treatments? The first step is to get people talking to each other—epidemiologists, discovery scientists, trialists and clinicians. As with so many areas of medicine and science, silo working is prevalent (Editorial 2016), leading to lost opportunities to join up different phases of work, to learn from different perspectives, and to avoid waste, dead ends and duplication. Joint meetings, interdisciplinary research groups, and embedding science in clinical teaching at all phases of training, are all potentially useful initiatives. Improved communication will undoubtedly help to ensure a consistent approach to sarcopenia research from bench to bedside.

### Studying the right populations

If we really want to know about the epidemiology, pathophysiology and treatments for sarcopenia, we need to study people with sarcopenia—not related but distinct populations such as those with functional impairment. Only then can we have confidence in our insights, and an expectation that the findings will be relevant to a patient with sarcopenia. In addition, we need to embrace the multimorbidity that inevitably accompanies sarcopenia. Failure to do so will render our work irrelevant to patients and their clinicians. Clinical studies—even very early phase experimental medicine studies—need to enrol patients with multimorbidity, as the presence of these other disease states may underpin, or at least modify, the phenotypic expression of sarcopenia. An example would be the role of chronic inflammation, posited to be important in sarcopenia. Many diseases (e.g. chronic obstructive pulmonary disease, atherosclerotic disease) are implicated in chronic inflammatory states, and a full understanding of how chronic inflammation contributes to sarcopenia requires study in patients with diseases that contribute to that chronic inflammatory state.

### What would an ideal intervention look like?

Next, we need to consider what a good intervention for sarcopenia would look like. Selection of interventions for study needs to be informed by plausible biological mechanisms, but other considerations are important in selection at an early stage in the translational pathway. Low cost, ease of administration (ideally once every few months, but certainly not multiple times a day), ability to formulate an oral preparation, and a lack of adverse effects are all important factors.

Perhaps the most valuable property for candidate interventions in older people however is that of pleiotropic benefit. This goes rather against the current paradigm for intervention development, where multiple interventions are developed for each single disease state (Witham and George 2014). This paradigm has led to the problem of polypharmacy—multiple diseases require multiple medications, each one of which carries a range of side effects and drug interactions. Unsurprisingly, polypharmacy is associated with high rates of adverse events, many of which are attributable to medication use and not just to the underlying disease states. For many older patients, fewer, not more medications may be needed to improve overall health and quality of life (Scottish Government 2018). Medications with pleiotropic benefits are likely to be those that target fundamental biological processes of ageing that underlie multiple disease states. Such interventions thus have the potential to provide beneficial effects across several diseases from a single agent. To a limited extent, such agents do exist already—beta blockers are used to treat heart failure, hypertension and atrial fibrillation for example; and other candidate pleiotropic agents (e.g. allopurinol, metformin) are under investigation (Barzilai et al. 2016; Mackenzie et al. 2016). Candidate interventions for sarcopenia that also benefit bone health, cardiovascular health and other organ systems are a goal worth working towards.

There is plenty of scope here for different phases of the research process to inform each other—the results of clinical trials or observations can suggest new targets to research, just as fundamental biological research can help to both inform the choice of interventions to test and the characteristics of patients most likely to benefit from a particular intervention. Such a virtuous circle however requires clinicians, clinical researcher and discovery scientists not only to talk to each other, but to learn each others’ languages and cultures (Coller 2008).

### Selecting the right outcomes

Outcomes in clinical studies need to be informed by what patients, but also clinicians, health and social services deem important. This too has an impact on preclinical science—for instance if an intervention is found to improve muscle mass but not strength in preclinical work, the case for translating these findings into even early clinical studies is likely to be weak, and alternative approaches should be researched. Different types of outcomes are however likely to be appropriate for different phases of trial—early phase trials are likely to need to focus on measures relevant to the biological target of interventions, intermediate phase trials on physical performance, but later phase trials need to focus on physical function in daily life, together with outcomes such as falls, healthcare utilisation and the need for social care, as has been argued elsewhere (Kilsby et al. 2017). Finding reliable surrogate biomarkers that predict longer-term response to treatment in sarcopenia trials is also essential if we are to deliver cost-effective, early-phase trials in a timely manner.

## Building the capacity to deliver clinical research in sarcopenia

Finally, we need to address the challenges to finding and recruiting older people with sarcopenia into trials and other clinical studies. Efficient pathways to recruitment are needed, which are likely to be multistep, involving targeted searches of electronic databases, followed by prescreening [e.g. using the SARC-F questionnaire (Malmstrom and Morley 2013)]. Given the difficulties inherent in this process, the creation of registries of patients with sarcopenia is an attractive solution; one that is now being explored both in the UK and in mainland Europe. Such registries would comprise detailed phenotypic data including muscle mass, strength and physical performance, together with pre-consent from patients to be contacted when research that is suitable for them to participate in becomes available. Such an approach has worked well for other musculoskeletal diseases (Thompson et al. 2017) and may be a way to overcome many of the hurdles to rapid, efficient trial recruitment in sarcopenia. The limited life and health expectancy of those with sarcopenia presents the disease registry model with a challenge however; such registries will require constant recruitment effort to replace people who die or become to unwell to participate.

Such registries on their own will not be enough though. We also need to build networks of research-engaged clinicians to help recruit to, and run, clinical studies, to fund such studies adequately, and to develop more trialists with the expertise to design and manage trials for older people. Engaging clinicians in the research process will also help to ensure that research results are taken up into clinical practice more quickly. All of this will require investment as well as training, but this final piece of the jigsaw is essential if the knowledge that we are now accruing from discovery science in musculoskeletal ageing is to translate successfully to better treatment for sarcopenia.
